# The Procalcitonin And Survival Study (PASS) – A Randomised multi-center investigator-initiated trial to investigate whether daily measurements biomarker *Procalcitonin *and pro-active diagnostic and therapeutic responses to abnormal Procalcitonin levels, can improve survival in intensive care unit patients. Calculated sample size (target population): 1000 patients

**DOI:** 10.1186/1471-2334-8-91

**Published:** 2008-07-13

**Authors:** Jens-Ulrik Jensen, Bettina Lundgren, Lars Hein, Thomas Mohr, Pernille L Petersen, Lasse H Andersen, Anne Ø Lauritsen, Sine Hougaard, Teit Mantoni, Bonnie Bømler, Klaus J Thornberg, Katrin Thormar, Jesper Løken, Morten Steensen, Peder Carl, J Asger Petersen, Hamid Tousi, Peter Søe-Jensen, Morten Bestle, Søren Hestad, Mads H Andersen, Paul Fjeldborg, Kim M Larsen, Charlotte Rossau, Carsten B Thomsen, Christian Østergaard, Jesper Kjær, Jesper Grarup, Jens D Lundgren

**Affiliations:** 1 Dept. Of Clinical Microbiology 445, Hvidovre Hospital, Kettegård Allé 30, DK-2650, Hvidovre, Denmark; 2 Centre for Viral Diseases/KMA, Rigshospitalet, 2100 Copenhagen Ø, Denmark and Copenhagen HIV Programme, University of Copenhagen, Faculty of Health Sciences, The Panum Institute/Building 21.1, Blegdamsvej 3B, 2200 Copenhagen N, Denmark; 3 Dept. of Intensive Care Y13, Glostrup University Hospital, Nordre Ringvej 69, DK-2600, Glostrup, Denmark; 4 Dept. of Intensive Care I-303, Gentofte University Hospital, Niels Andersens Vej 65, DK-2900, Gentofte, Denmark; 5 Dept. of Intensive Care 542, Hvidovre University Hospital, Kettegaard Alle 30, DK-2650, Hvidovre, Denmark; 6 Dept. of Intensive Care 104, Herlev University Hospital, Herlev Ringvej 75, DK-2730, Herlev, Denmark; 7 Dept. of Intensive Care, Hillerød University Hospital, Helsevej 2, DK-3400, Hillerød, Denmark; 8 Dept. of Intensive Care, Aarhus University Hospital Skejby, Brendstrupgaardsvej 100, DK-8200, Aarhus N, Denmark; 9 Dept. of Intensive Care, Aarhus University Hospital Århus, Nørrebrogade 44, DK-8000, Aarhus C, Denmark; 10 Dept. of Intensive Care, Roskilde University Hospital, Køgevej 7, DK-4000, Roskilde, Denmark

## Abstract

**Background:**

Sepsis and complications to sepsis are major causes of mortality in critically ill patients. Rapid treatment of sepsis is of crucial importance for survival of patients. The infectious status of the critically ill patient is often difficult to assess because symptoms cannot be expressed and signs may present atypically. The established biological markers of inflammation (leucocytes, C-reactive protein) may often be influenced by other parameters than infection, and may be unacceptably slowly released after progression of an infection. At the same time, lack of a relevant antimicrobial therapy in an early course of infection may be fatal for the patient. Specific and rapid markers of bacterial infection have been sought for use in these patients.

**Methods:**

Multi-centre randomized controlled interventional trial. Powered for superiority and non-inferiority on all measured end points. Complies with, "Good Clinical Practice" (ICH-GCP Guideline (CPMP/ICH/135/95, Directive 2001/20/EC)). Inclusion: 1) Age ≥ 18 years of age, 2) Admitted to the participating intensive care units, 3) Signed written informed consent.

Exclusion: 1) Known hyper-bilirubinaemia. or hypertriglyceridaemia, 2) Likely that safety is compromised by blood sampling, 3) Pregnant or breast feeding.

Computerized Randomisation: Two arms (1:1), n = 500 per arm: Arm 1: standard of care. Arm 2: standard of care and Procalcitonin guided diagnostics and treatment of infection.

Primary Trial Objective: To address whether daily Procalcitonin measurements and immediate diagnostic and therapeutic response on day-to-day changes in procalcitonin can reduce the mortality of critically ill patients.

**Discussion:**

For the first time ever, a mortality-endpoint, large scale randomized controlled trial with a biomarker-guided strategy compared to the best standard of care, is conducted in an Intensive care setting. Results will, with a high statistical power answer the question: Can the survival of critically ill patients be improved by actively using biomarker procalcitonin in the treatment of infections? 700 critically ill patients are currently included of 1000 planned (June 2008). Two interim analyses have been passed without any safety or futility issues, and the third interim analysis is soon to take place. Trial registration number at clinicaltrials.gov: Id. nr.: NCT00271752).

## Background

### 1.1. Sepsis in the Intensive Care Unit

Sepsis caused by bacteria is the major cause of mortality in ICU-patients. Rapid initiation of the correct treatment is of crucial importance for the probability for surviving the condition [1,2].

Bacterial sepsis in ICU patients is a condition that 1) is complex to diagnose, 2) hard to monitor regarding treatment effect, 3) very frequent in these patients and 4) carries a high mortality risk for the patient, if treatment is not adjusted precisely and rapidly, when treatment failure is eminent.

Reasons for this can be:

1) Symptoms are rarely expressed, since many ICU.patients are unconscious.

2) Clinical findings have often got an atypical appearance because of an immunological incompetence and because of the treament regimens (dialysis, intropic drugs a.s.o.).

3) The established infection markers, Leucocytes and C-reactive protein, are influenced by many other conditions than bacterial infection, and they are released to the blood steam with a considerable delay of time.

Specific and rapid markers for sepsis have therefore been sought for monitoring of ICU-patients through many years.

#### Procalcitonin as a marker for Sepsis

Serum-Procalcitonin (PCT) is a sensitive and specific biomarker for bacterial sepsis, and the level of PCT increase is correlated to the severity of the infection [3,4]. It is documented that PCT release to the blood stream is initiated 2–6 hours after bacteria or bacterial products are presented in the blood [5], which is faster than the established markers. The PCT level increases transiently, (in opposition to C-reactive protein (CRP) and leucocytes) for only 12–24 hours after surgery, if hvis the patient is not developing an infection. If the PCT level increases for more than 24 hours after surgery, bacterial sepsis should be suspected [6,7]. Our group has earlier shown that the mortality rate in ICU patients is increased markedly from day-to-day, when the PCT level increases [8]. Approximately 10.000–15.000 patients are admitted to ICU's in Denmark every year. 3000–4000 of these die in connection to this ICU admittance and a large part of these deaths are attributable to bacterial sepsis (assumed from numbers from the Grand Capital Region).

Globally, no randomized interventional studies have been conducted with the aim to reduce mortality in the ICU by guiding the treatment of infections with daily PCT measurements.

Followingly, there is at the present time, not satisfying evidence to implement PCT-measurements in the everyday practice in ICU-departments [9].

### 1.2. Study Aim

#### Primary

aim: To investigate whether a treatment strategy, that is guided by daily measurements of the infection biomarker "Procalcitonin" can reduce the mortality rate in intensive care unit (ICU) patients. Apart from this, secondary aims concern reducing several early and late complications to infection, hospitalization and antibiotic comsumption.

### 1.3. Hypothesis

Daily measurements of the infection biomarker Procalcitonin can precisely and in a timely manner monitor the emergence and the course of a serious infection in ICU patients and can via rapid and sufficient changes in the diagnostics and therapeutics increase the survival rate.

## Methods

### 2.1. Design and sample size

Multi-centre randomized controlled single blinded interventional trial. Powered for superiority and non-inferiority on all measured end points. Complies with, "Good Clinical Practice" (ICH-GCP Guideline (CPMP/ICH/135/95, Directive 2001/20/EC)) and all applicable regulatory requirements.

Initially the patient is randomized either to "PCT-guided treatment" or "The Control Group".

All included patients have blood samples made every morning (including weekends, national holidays etc.).

#### Sample size

With a sample size of 500 per group and an assumed mortality rate of 25% in the control group and 17.5% in the PCT group there will be 80% probability that a negative result (Confirming the Null Hypothesis) is true. At the same time there will be < 5% probability of falsely declaring the alternative hypothesis correct. [Power 80%, stringency 5%].

The sample size calculation is made in cooperation with Dep. of Statistics, UCLA and Jim Neaton, Ph.D. Professor, Division of Biostatistics, Biometry, University of Minnesota, both USA.

The Study Design has been chosen from its strength to investigate the study aim. The Study Group has a large and thorough experience with randomized interventional trials, since the initiating powers with Professor Jens D. Lundgrens as a leading power has lead and completed several randomized controlled trials, and members of the study group are presently engaged in other studies with this design.

### 2.2. Patients

#### Inclusion

A subject will be eligible for inclusion in this trial only if all of the following criteria apply: 1) Male or female, aged ≥ 18 years of age, 2) Admitted to the participating intensive care units. Patients should be included within 24 h. If a patient has not been included at this time, this patient cannot be included in the present admittance, 3) Subjects should in the investigator's opinion be likely to be admitted to the ICU for more than 24 h. Subjects should not be likely (<10%) to die or be discharged in this period of time, 4) Ability to understand and provide written informed consent to participate in this trial, or ability to understand and provide oral informed consent in presence of at least one impartial witness who should sign and personally date the consent form or the subject's legally acceptable representative can understand and provide written informed consent if the subject is not capable of this because of the present mental or physical condition of the subject.

#### Exclusion

A subject will **NOT **be eligible for inclusion in this trial if any of the following criteria apply:

1) Subjects with known hyper-bilirubinaemia (>0.4 mg/ml) or hypertriglyceridaemia (>10 g/l) since this can interfere with measurements. If subjects with unknown status on these points are included and have PCT measurements, the measuring-equipment will detect these conditions, 2) Subjects suffering from a blood disorder, where daily sampling of 7 ml of blood for maximally 28 days (210 ml distributed on 28 days) will be an inconvenience or a potential risk, which could compromise the safety of the subject.

### 2.3. Scientific participants, organisation

The study group consists of physicians from all the participating ICU's, Dept.'s of Clinical Microbiology and from specialists in infectious medicine. The study group has a solid research experience, with numerous publications in the best international journals.

### 2.4. Randomization and blinding

The randomisation is performed via computer, on-line to the PASS study centre and is stratified according to site, age and initial Acute Physiology And Chronic Health Evaluation II (APACHE II) score. For patients randomised to the PCT intervention arm, daily PCT levels are communicated to the team responsible for the clinical management together with a recommendation of what interventions the investigator team is expected to initiate based on the PCT measurement. In the control arm, blood samples for PCT will be analysed simultaneously with samples from the PCT intervention arm, but results of these PCT analyses will remain blinded for the investigators until the study has been completed. The PCT measurements will be conducted daily as long as the patient is admitted to the ICU, but maximally 28 days from time of enrolment in this study. Patients transferred from one ICU to another ICU, will remain in the trial provided that the receiving ICU also participates in this trial.

### 2.5. Treatment and Intervention

The aim of the PCT guided treatment is to reduce time to relevant treatment of a serious infection and thereby to reduce the mortality. All subjects will receive the standard-of-care evaluations and therapeutic interventions recommended in the ICU at which the patient is admitted to. Subjects in the PCT measurement group will additionally receive expanded diagnostics and treatment should the PCT levels be found to high and/or increasing (see section 3.3.1 for definitions).

Access to results of PCT measurements of any kind (semi-quantitative or quantitative) at any time in the study period is not allowed for patients randomised to the control arm.

The PASS study group in collaboration with the PASS Steering Committee, will issue guidelines for the composition of the interventions that a high or increasing PCT level would mandate. Some variation between sites is acceptable, whereas all patients within a given ICU should follow that ICU's guidelines. The guidelines will be updated when new information becomes available. In the guidelines, there may be several alternatives indicated for a given situation. The investigator is not mandated to follow the guidelines.

#### Antimicrobial interventions – principles

The investigator or an intensive care physician, who has been sufficiently instructed in all aspects of the trial, must prescribe all antimicrobial drugs that are prescribed on basis of an increasing PCT. The investigator must check for possible drug-drug interactions between any of the drugs prescribed guided by PCT changes and other agents that may be metabolised via the same enzyme systems or organs. To assist the investigator, information on this topic is included in the Manual of Operational Procedures. Also, the product label of each drug prescribed should be reviewed.

General principles that will be followed regarding antimicrobial therapy of sepsis are:

▪ Antimicrobial agents are prescribed, when possible, according to the resistance pattern of the causative microorganism.

▪ When the causative microorganism is not known, antimicrobial agents are prescribed according to knowledge of which microorganisms normally and possibly infect the suspected focus.

▪ When neither the microorganism nor the focus of infection is known, one or more broad-spectrum antimicrobial agents are selected. If the effect is not sufficient, the spectrum of the used antimicrobial agents is additionally expanded, often with anaerobic active agents, gram-positive active agents and antifungal agents. Conversely, if the effect is sufficient, the spectrum of used antimicrobial agents is narrowed according to knowledge of focus and causative microorganism.

▪ In empiric sepsis treatment, a combination of a β-lactam/Carbapenem + a fluor-quinolone is chosen if not contra indicated in the specific subject. This treatment can be supplemented with nitroimidazoles, glycopeptides, oxazolidinones and azoles. More specific treatment regimes are initiated and guided by findings regarding the causative microorganism and/or focus of infection.

▪ Dosages of antibiotics are decided according to the recommendations of the specific ICU.

The toxicity management guidelines detailed below refer to all components of the antimicrobial treatment used in the trial.

#### Overdose and Toxicity

Antimicrobial agents may be interrupted because of the development of adverse events (AE) at the discretion of the investigator and according to the severity of the AE. The dose of all antimicrobial drugs may be reduced, interrupted or reintroduced according to standard practice at the time, and depending on the severity of the AE.

Subjects who require a dose modification should be re-evaluated on a daily basis.

The investigator is responsible for taking appropriate precautions to ensure that the risk of developing toxicity is minimised.

#### Procalcitonin levels and diagnostic and therapeutic consequenses

The situation mandating additional interventions in the the PCT intervention arm is based on the following criteria: → PCT levels > 1.00 ng/ml and the PCT level increases one day to the next or has an "irrelevant" decrease of < 10%. The daily assessment of PCT guided interventions will be as follows:

A) Subjects with PCT levels > 1.00 ng/ml based on the first determination after enrolment into the study will follow the principles for interventions as detailed below.

B) Subjects with PCT levels > 1.00 ng/ml and with a day (n) to day (n+1) PCT increase or a decrease of < 10% (irrelevant decrease) will follow the principles for interventions as detailed below.

1) Microbiology: blood cultures, airway cultures, urine cultures and samples from any other suspected foci, 2) Considerations of whether to perform diagnostic imaging: one or more of the following: Chest X-ray, Ultra-sonic examination of suspected focus, Computerised Tomography of relevant areas, Magnetic Resonance imaging of relevant areas, other imaging techniques, 3) Surgical drainage of possible un-drained foci, 4) Antimicrobial therapy expansion. Treatment will be guided by any relevant findings: microbial or diagnostic imaging, or other findings. If focus and microorganism of infection is not clear, steps will be: A) Empirical sepsis treatment, B) Empirical sepsis treatment with anaerobic and gram positive coverage, C) Empirical sepsis treatment with anaerobic and gram positive coverage and/or fungal treatment.

Subjects with PCT levels < 1.00 ng/ml will continue to receive standard-of-care. Subjects with PCT levels >1.00 ng/ml and with a day-to-day PCT decrease of > 10% will continue to receive standard-of-care.

**Figure 1:**
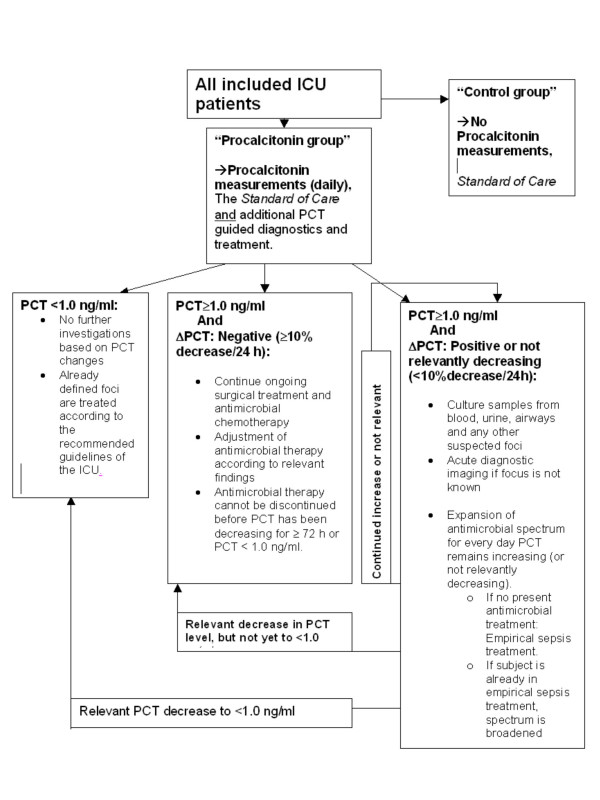
**Strategy for dynamic Procalcitonin-guided treatment.** According to the kinetic/elimination prophile of procalcitonin, a decrease of at least 10% pr. 24 h should be observed, when bacterial infection is under control.

Precise guidelines for this (antimicrobial) treatment will be made specifically for every ICU in concordance with the local choices regarding antimicrobial agents. For PCT guided diagnostics and treatment algorithm, see Fig 1.

#### Daily PCT analizing results

PCT-analysis is delivered before 11 A.M every morning, but only for patients in the "PCT-guided group", (i.e. not control group). When the PCT answer is delivered, all PCT-guided patients are set in a "PASS-category". This category carries a specific diagnostic and therapeutic programme, aimed on improving the treatment of an emerging infection, where there is treatment failure. In this way, as hypothesized, the treatment is expanded to cover the causative micro organism, even before the patient comes to suffer from organ failure, circulatory collaps etc.

### 2.6. Data collection

Data is collected in Case Report Forms (CRF), which are specific for every patient. CRF's are quality controlled by an internal as well as an external control organisation, and is then entered into the PASS-database. For overview of data collection, see table 1.

**Table 1:** Data registration at time points in the patient course in the intensive care unit.

Evaluation	Day (screening & baseline)			Day (counting after admission to ICU) (follow-up)				
	1	2 thru 28 or dis-charge	Day = Discharge/death	28	30	60	90	180

Informed Consent	X							
Entry Criteria	X							
Demography	X							
APACHE II/SOFA	X	X	X					
Infections during this hospital admission	X	X						
Current medical conditions	X	X	X					
State of daily function and health	X				X			X
Mortality		X	(X)	X		X	X	X
PCT	X	X	X					
AUC_procalcitonin_			X					
Concurrent Medications^a^	X	X	X		X	X	X	X
Haematology	X	X	X					
Clinical chemistry	X	X	X					
Adverse events	X^a^		X					
Serious Adverse Events	X^a^	X	X		X	X	X	X

All data are registrered *real-time *in case report forms and according to the Good Clinical Practice legislation and guidelines. Clinical and laboratory Evaluations

a Adverse events and serious adverse events are registered daily

THIS PROTOCOL IS A SHORT AND MODIFIED EDITION FOR PUBLICATION: THE FULL LENGTH PROTOCOL (41 pages) WITH ALL REFERENCES CAN BE REQUIRED UPON REQUEST FROM THE AUTHORS.

#### Pre-entry Evaluations

The site must obtain subject consent in the form of a written informed consent form prior to the initiation of any pre-entry procedures as outlined in this protocol. The consent form must be approved by the IEC of each participating site. The pre-entry evaluation will be conducted the first day of the trial by an investigator in the ICU and will include an evaluation of whether the patient fulfils the requirements for enrolment in this trial.

Subjects who fail to meet the entry criteria may not be re-screened for this protocol until 28 days after the failed pre-entry evaluation. Hence, enrolment of such patients will require that the patient is re-admitted to the ICU after at least 7 days outside of the ICU after the time of the first screening.

#### Baseline (Day 1) Evaluations

The following evaluations should be performed at baseline (Day 1):

Note: For this trial, Baseline (Day 1) is defined as the day on which the subject has his/her first blood sample for PCT measurement. The following data are to be collected on day 1: A) Demography including date of birth, weight, height, and indication for admittance to the ICU, B) Infections found in the subject in this hospital admission prior to admittance to the ICU, C) Present infection focus/etiologic microorganism, D) APACHE II score (Temperature, Mean Arterial Pressure, Heart Rate, Respiratory Rate, FIO2, HCO3-, pH (arterial), Se- Na+, K+, Creatinine, Haematocrite, White Blood Count+ differential count, Glasgow Coma Scale) E) Current medical conditions, F) Pre-admittance daily function and health state, G) Professional career 1) Student, 2) Part time work, 3) Full time work, 4) Early retirement, 5) Retired. H) Health: 1) Congenital handicapped, 2) Acquired handicap, 3) Chronic disabling disease, 4) Chronic non-disabling disease, 5) Healthy, I) Self-supportance: 1) Lives in nursing home, 2) Lives in a flat connected to a nursing home, 3) Own home with external help ≥ once/day, 4) Own home with external help < once daily, 5) Own home, no help required, J) Hospital need: 1) ≥ 3 months admitted to a hospital/last year, 2) 1–3 months admitted to a hospital/last year 3) 1–30 days admitted/last year, 4) No admissions, ambulatory visits ≥ 6/last year, 5) No admissions, ambulatory visits 1–5/last year, 6) No admissions, No ambulatory visits/last year, K) Adverse events/other complications to treatment given in this hospital admission (ongoing clinical conditions at Day 1 shall be recorded in the "Adverse Event and Medical Condition Form" of the CRF at this time, regardless of the fact that such conditions may not subsequently be found to fulfil the definitions for an adverse event, L) Haematology: haemoglobin, platelet count (WBC count mentioned as part of APACHE II), M) Clinical chemistry: Albumin, Bilirubin, Factor 2-7-9, Alanin Amino Transferase (ALAT)/Aspartate Amino Transferase (ASAT), Alcaline Phosphatase, Creatinine, Carbamide, Na+, K+, Phosphate, Ca2+, C-reactive protein (some are also mentioned as part of APACHE II), N) Baseline PCT: The daily PCT determination is done real-time at the Department of Clinical Biochemical Department, Hvidovre Hospital, using the EC-approved measuring instruments and reagents. For each subject, the same methodology should be used throughout the trial period. The KRYPTOR^® ^PCT BRAHMS sensitive assay is the accepted standard assay. Other licensed assays may be used instead if judged by the PASS steering committee to have a comparable performance compared to the indicated assay.

#### On Trial Evaluations

On trial assessments will be completed at the following time-points unless otherwise specified:

While admitted to the ICU, the following information will be registered unless specified otherwise:

Daily while patient is admitted to the ICU: A) Clinical signs of new (nosocomial) infections, B) Microbiological or radiological evidence of new (nosocomial) infection, C) Defined Day Doses of antimicrobial chemotherapy, D) APACHE II score (Temperature, Mean Arterial Pressure, Heart Rate, Respiratory Rate, FIO2, HCO3-, pH (arterial), Se- Na+, K+, Creatinine, Haematocrite, White Blood Count+ differential count, Glasgow Coma Scale), E) Occurrence of sepsis, severe sepsis, septic shock, DIC. Assessment of Glasgow Coma Scale, measurement of Blood Pressure (systolic blood pressure < 90), days with artificial ventilation, Factor 2-7-9 < 0.7, creatinine (increase factor 3 from baseline), MODS, F) Adverse events/other complications to treatment given in the ICU (ongoing clinical conditions at Day 1 shall be recorded in the "Adverse Event and Medical Condition Form" of the CRF at this time, regardless of the fact that such conditions may not subsequently be found to fulfil the definitions for an adverse event, G) Haematology: haemoglobin, platelet count WBC (WBC count also mentioned as part of APACHE II), H) Clinical chemistry: Albumin, Bilirubin, Factor 2-7-9, Alanin Amino Transferase (ALAT)/Aspartate Amino Transferase (ASAT), Alcaline Phosphatase, Creatinine, Carbamide, Na+, K+, Phosphate, Ca2+, C-reactive protein (some are also mentioned as part of APACHE II), I) Blood sample for PCT determination, J) Diagnostic imaging procedures performed, K) Non-routine microbiological sample taken, L) Surgical procedures performed, M) Change in antimicrobial chemotherapy, N) At the day of discharge from ICU or day of death or later: Mortality and time of death, and the cause hereof: AUCProcalcitonin (at discharge from the ICU, will remain blinded in the control arm), O) Discharge and post-discharge daily function and health state (obtained on day 30 and 180): Professional career: 1) Student, 2) Part time work, 3) Full time work, 4) Early retirement, 5) Retired, P) Health:1) Congenital handicapped, 2) Acquired handicap, 3) Chronic disabling disease, 4) Chronic non-disabling disease, 5) Healthy, Q) Self-supportance: 1) Lives in nursing home, 2) Lives in a flat connected to a nursing home, 3) Own home with external help ≥ once/day, 4) Own home with external help < once daily, 5) Own home, no help required, R) Hospital need: 1) ≥ 3 months admitted to a hospital/last year, 2) 1–3 months admitted to a hospital/last year 3) 1–30 days admitted/last year, 4) No admissions, ambulatory visits ≥ 6/last year, 5) No admissions, ambulatory visits 1–5/last year, 6) No admissions, No ambulatory visits/last year.

### 2.7. Interrim analyses

Interrim analyses are made at specified inclusion numbers (250, 500, 750) and a Data and Safety Monitoring Board elaborate the open reports from these. If an interim analysis gives reason for discontinuing the study from safety, efficacy or futility reasons, notion will be taken, and a procedure for taking this step is described in detail in the detailed protocol. Stopping the trial will not be based purely on a statistical decision but also on the recommendation of the DSMB.

### 2.8. Analyses and statistics

Efficacy and safety analyses are made on the "intention to treat"-group. The main analysis of the primary endpoint will be the comparison of the two differing treatment strategies, regarding 28-day-mortality. The stratified log-rank test and Kaplan-Meier estimates will be used. The treatment effect on reduction of early and late emrging complications to serious bacterial infection (severe sepsis, septic shock, disseminated Intravasulat Coagulation (DIC) and multi organ dysfunction syndrome will be determined as well as the ability of the strategy to reduce the consumption of antibiotics. The research unit, Copenhagen HIV Programme, which is the coordination centre, has two biostaticians in the staff.

### 2.9. Time plan/Status

Recruitment start and study registration:

703 patients have been included until now (July 2008). The PASS-study is registered at National Institutes of Health (NIH), USA at trial registration: clinicaltrials.gov, Id. nr.: NCT00271752. Recruitment of patients has started in 8 intensive care units (Jan, June, Oct. 2006, Jan + May 2007 × 4). Planned study closure, Oct/Nov. 2008.

### 2.10. rocalcitonin-analyses

For PCT-analysis, a fully automated and highly sensitive method, the, KRYPTOR^® ^PCT BRAHMS, has been chosen. The procedure is done every day in dept. of Clinical Microbiolgy at Hvidovre Hospital and at Skejby Hospital. When the answer to the analysis is ready, the answers are sent out to via the database to ensure blinding of PCT-analysis in the control-arm. An interpretation frame and corresponding treatment algorism is delivered to all ICU's at each nescessary intervention.

## Discussion

The PASS-Study is accepted by the relevant local and national institutions in Denmark, which regulate clinical research, so as the local Ethical Board, the Data monitoring authorities. The research group behind the PASS-study has completed a thorough ethical discussion regarding the main ethical aspects in running randomized trials, withholding potentially beneficial treatment for control group patients, conducting interventional trials on unconscious patients and aspects regarding the need to conduct the trial at this point in time.

Additionally the question of how a bio-bank should be made in the ethically most correct way has also been discussed.

Main points from these discussions are noted in the detailed protocol.

### Adverse events (AE)

Adverse events will be tabulated by treatment group, maximum intensity, attributability to various antimicrobial agents and by seriousness. Treatment related adverse events that lead the subject to prematurely discontinue one or more of the originally prescribed antimicrobial agents will also be summarised.

Clinical chemistry and haematology results will be presented by summary statistics and quartile plots of measured results. Change from baseline for these results will also be presented. Baseline is defined as the laboratory data collected at Day 1 (before the first blood sample for PCT analysis). Subjects must have both a baseline and an "on treatment" measurement to be included in the change from baseline analysis.

Treatment emergent toxicity grades will be presented for each graded laboratory parameter by treatment group. A graded toxicity is considered treatment emergent if it develops or increases in intensity, post Day 1. Treatments will include established and approved antimicrobial treatments, which are already used daily in the participating ICU's.

Concurrent medications and blood products will be summarised by randomised treatment group.

### Adverse events (AE) and Serious Adverse Events (SAE) – Discussion

As mentioned other places in this protocol, the direct inconvenience for subjects in this study is sampling of 7 ml of whole blood daily in the same session as the routine blood samples are made, every morning. Therefore it is reasonable to expect that AE's and SAE's as a direct consequence of this blood sampling will not occur. Indirect AE's as a consequence of potential overly treatment are likewise not likely to occur according to the available literature on the issue, especially because the most striking result of the previously published RCT's is a reduction of antibiotic exposure in the PCT-guided group. All interventions, that are performed in this study are well-known, thoroughly tested and accepted treatments, so it does not seem reasonable to apply the same procedures for this study regarding AE's as e.g. a study where a new drug is to be assessed for safety (or effect).

Investigators will, however, have the opportunity to report events, that they find unexpected, in the Case Report Form (CRF). In this part of the CRF, it is possible to classify unexpected events in groups of "relatedness" to the antimicrobial treatment as "no relation", "unlikely relation", "possibly related", "probably related" or "definitely related.

### Serious unexpected events or unexpected events

Serious inexpected events and unexpected events, that can be related to the antimicrobial treatment will in both treatment groups be reported to the Danish Medicines Agency "Lægemiddelstyrelsen" according to the Danish legislation on this point.

The primary and the secondary endpoints that are registered daily in the case report form are all adverse events or serious adverse events, i.e. death, complications to sepsis, increased antibiotic exposition and prolonged hospital stay. These are registered routinely and daily in the part of the CRF dealing with effects of the treatments. At inclusion, all patients in the study are threatened by potentially lethal illnesses.

### Scientific Perspective

Numerous observational studies argue, that there is a possible diagnostic and therapeutic value of PCT-measurments at bacterial sepsis in the ICU, and implementation of the method for bacterial sepsis has therefore been discussed for several years. No large scale interventional studies have been conducted with an ICU patient population. If PCT measurements where to be implemented in all ICU's in Denmark, the laboratory costs alone will carry an expense per year of 800.000 € – 2.5 million €. This is a large expense to add on for the health system, without the suffient evidence for an effect of the method. At present, there is not high-level evidence (level 1–2) to support implementation of PCT measurements in the ICU, and at the same time, there is not high-level evidence to refrain from implementing this biomarker.

One of the following four situations will occur, if evidence at a higher level is not obtained: 1) PCT measurements are not implemented in the ICU, in spite of a clinically significant effect for the patient, 2) PCT measurements are not implemented in the ICU, and there is no clinically significant effect for the patient, 3) PCT measurements in the ICU are in fact implemented, in spite of no clinically significant effect for the patient, 4) PCT measurements in the ICU are in fact implemented, and there is, in fact, a clinically significant effect for the patient.

Situations 1 and 3 above will have large negative consequences for patients (1) or society (3). At present, there is no evidence to reveal which situation will occur, since no randomised controlled trials have been conducted to show if mortality in critically ill patients can be reduced by using a strategy of daily standardised Procalcitonin measurements as an early detector of serious bacterial infection.

If the PASS-study is concluded as planned, situations 2 or 4 can be chosen on the basis of evidence level 1 background.

### Database and biobank

The PASS-Study will furthermore give rise to a unique intensive care research database and biobank with 1000 patients, and blood samples frozen from every day of the admission period on all of these patients. This material will have a statistical and scientific power to conclude on many hypotheses regarding the diagnostics and treatment of critically ill patients.

## Competing interests

The Procalcitonin And Survival Study is an investigator initiated and driven study. The participating investigators in the Steering Committee (no company-members) have the right to the results and no other parties have rights to results. No other parties than the steering committee has or will have any rights regarding the design or conduct of the study or substudies made via the biobank and database.

JUJ has fulfilled speaking engagements and received payments for this from the Brahms company, that makes kits for procalcitonin analysis. All other authors and contributors declare no conflicts of interest or competing interests.

## Authors' contributions

J–UJ, BL, LH, TTM and JDL designed, wrote the protocol and initiated the study. PLP, LHA, AØL, SH, TTM, BB, KJT, KT, JL, MS, JAP, HT, PS–J, MB, SH, MHA, PF, KML, CR, CBT, CØ, PC, JK, JG, J–UJ, BL, LH, TM and JDL managed the trial, included patients and collected data. All authors amended and approved on the final manuscript.

## Pre-publication history

The pre-publication history for this paper can be accessed here:


